# Healthcare professional perspectives on barriers and enablers to falls prevention education: A qualitative study

**DOI:** 10.1371/journal.pone.0266797

**Published:** 2022-04-27

**Authors:** Hazel Heng, Debra Kiegaldie, Susan C. Slade, Dana Jazayeri, Louise Shaw, Matthew Knight, Cathy Jones, Anne-Marie Hill, Meg E. Morris

**Affiliations:** 1 Academic and Research Collaborative in Health, La Trobe University, Bundoora, Victoria, Australia; 2 Faculty of Health Science, Youth & Community Studies, Holmesglen Institute, Melbourne, Victoria, Australia; 3 Eastern Health Clinical School, Monash University, Melbourne, Australia; 4 The Victorian Rehabilitation Centre, Healthscope, Glen Waverley, Victoria, Australia; 5 School of Allied Health, WA Centre for Health & Ageing, University of Western Australia, Perth, Western Australia, Australia; 6 Physiotherapy, College of Healthcare Sciences, James Cook University, Townsville, Queensland, Australia; University of South Australia, AUSTRALIA

## Abstract

In hospitals, patient falls prevention education is frequently delivered by nurses and allied health professionals. Hospital falls rates remain high globally, despite the many systems and approaches that attempt to mitigate falling. The aim of this study was to investigate health professional views on the enablers and barriers to providing patient falls education in hospitals. Four focus groups with 23 nursing and allied health professionals were conducted at 3 hospitals. Three researchers independently coded the data and findings were analysed thematically with a descriptive qualitative approach to identify and develop themes according to barriers and enablers. Barriers included (i) limited interprofessional communication about patient falls; (ii) sub-optimal systems for falls education for patients and health professionals, and (iii) perceived patient-related barriers to falls education. Enablers to providing patient falls education included: (i) implementing strategies to increase patient empowerment; (ii) ensuring that health professionals had access to effective modes of patient education; and (iii) facilitating interprofessional collaboration. Health professionals identified the need to overcome organisational, patient and clinician-related barriers to falls education. Fostering collective responsibility amongst health professionals for evidence-based falls prevention was also highlighted.

## Introduction

Health professionals have a key role in mitigating falls in hospitals [1, 2]. This includes delivering multi-factorial interventions that include patient education, clinician training, rehabilitation, exercises, environmental modifications, and optimal hospital falls systems and policies [3–6]. Of these, patient education is key and has evidence for effectiveness for falls prevention in hospitals [7, 8]. Nurses and allied health professionals deliver falls prevention education using face-to-face discussions with individuals or groups, brochures, pamphlets, posters, patient education and videos [9].

Hospital falls continue to be a challenging issue worldwide, ranging from 6 to 17 falls per 1000 bed days [8, 10, 11]. When patients fall, they are at risk of sustaining injuries such as fractures, intracranial bleeding and lacerations, which may lead to further complications [12, 13]. People over the age of 65 years and those over the age of 50 years with a co-morbidity are at greater risk [14]. Risk factors for hospital falls include advanced age, a previous history of falls, cognitive impairment and multi-morbidity [15, 16].

To empower patients to prevent falling whilst in hospital, a patient-centred approach can be used [17, 18]. Engaging patients in the decision making processes about their own health and wellbeing makes them more likely to adhere to recommended falls prevention strategies [19, 20]. The content of the falls education program and mode of delivery also require careful consideration [21]. Many previous studies on patient falls education in hospitals did not take into account educational design or the quality of the education provided to patients [9]. When this was done effectively, patients and health professionals were empowered to prevent falls [2, 8, 22]. Patient falls education was shown to improve patient knowledge and awareness of falls mitigation strategies, as well as health professional motivation for delivering education [2, 22]. Well-designed education has the potential to empower patients by increasing self-efficacy and encouraging them to engage with falls prevention strategies [22].

Since nursing and allied health professionals are the main providers of falls prevention education in hospitals, it is imperative for them to deliver patient education effectively, with consideration to evidence-based approaches. While there have been studies of health professional experiences with patient falls in hospital [23, 24], and health professional views of patient education [25, 26], there has not yet been a study exploring health professional views about hospital patient falls education. A greater understanding of health professional views can help to facilitate implementation of evidence-based practice [27, 28]. Identifying health professional views is necessary to facilitate a process where their needs are met and therefore lead to them being more willing to engage with implementing evidence-based approaches [29, 30]. Aligning evidence-based strategies with their views is likely to foster more engagement and adherence [31]. To reduce hospital falls rates, it is important to understand health professional beliefs about how to design and deliver effective patient education.

The aims of this study were to: (i) explore health professional views of the enablers and barriers to delivering evidence-based patient falls prevention education programs in hospitals; and (ii) understand health professional perceptions of appropriate educational delivery for hospitalised patients.

## Methods

### Design

Semi-structured focus groups with nursing and allied health professionals were conducted at three large Australian hospitals. To gain a deep understanding of health professional views and experiences of hospital falls prevention education, an explorative and descriptive qualitative design was selected [32, 33]. Thematic analysis was used to identify patterns and ideas across participant data [32, 34]. A descriptive qualitative approach to data analysis allowed major themes to be captured [17]. A focus group design was chosen as it allowed for new insights that can arise from participant interactions [35, 36].

### Recruitment and sampling

A purposive sampling methodology was used to recruit participants at one acute and two rehabilitation hospitals. The research team liaised with allied health managers and nurse unit managers for recruitment and the managers distributed participant information statements to potential participants. Participants were reassured that there was no compulsion for them to participate and declining to participate would not disadvantage them in any way.

Registered nurses or allied health professionals were eligible to participate if they worked in hospitals. Health professionals who worked in non-inpatient settings were excluded. Prior to commencing the focus groups, the health professionals completed a questionnaire on their occupation, main area of work, clinical experience, and qualifications.

### Data collection

Once informed consent was obtained, focus groups were conducted at a convenient time for the stakeholders at each hospital site. The interview questions were developed with expert advice from members of the study team and after considering the analysis from a scoping review (Table 1) [9]. The interview aimed to obtain health professional views on the enablers and barriers to evidence-based patient falls education. Interviews were semi-structured with the interviewer using the list of questions as a guide. Each focus group interview was audio-recorded and transcribed verbatim by a third-party transcription service. To ensure anonymity, each participant was de-identified with a number used in all transcripts and questionnaires.

**Table 1:** Interview question guide for focus groups.

1. Why do you think patients fall in hospital?
2. Have you provided falls prevention advice or information to patients?
a) If yes, where and when? How do you provide it? What information do you give?
b) If no, why not?
3. What makes it easy to deliver falls prevention education? Why?
4. What makes it difficult to deliver falls prevention education? Why?
5. Do you think falls prevention education will prevent or reduce falls?
6. What do you think will help patients follow and engage with patient education?
7. What do you think about the current methods of hospital falls prevention education and policies?
8. Do you have any suggestions for changes in falls prevention practice in hospitals?

An experienced qualitative researcher (SCS) facilitated the focus groups and another researcher (HH) took notes to capture additional observations and non-verbal responses. Members of the research team conducted data analysis (HH, DK, SCS, MEM), and provided clinical expertise and advised on research design and implementation (MEM, DK, DJ, MK, CJ, A-MH, LS). None of the research team had associations with the participants. All researchers had a background in healthcare which may result in pre-existing views about barriers and enablers to hospital falls prevention. These views were acknowledged and carefully managed by reflection and discussions in meetings during data collection and analysis.

### Data analysis

The data were analysed thematically to identify patterns and develop themes [32]. Following the transcription of audio-recordings, the audio files and transcribed documents were compared to ensure accuracy. Three researchers (SCS, HH and DK) independently coded the data and identified categories and preliminary themes using a template based on the structure of the interview questions that were focused on barriers, enablers, and educational delivery. An iterative audit trail to ensure rigour of the analysis was undertaken. Findings were discussed and documented at each stage via video-conferencing or face-to-face meetings. Spreadsheets, documents, and tables were used to present coded data. If there were differences in opinion, another researcher (MEM) was consulted to achieve consensus. Participant quotes were used to generate final themes and sub-themes. Some examples are provided in the textboxes in the results section of this manuscript.

To minimise the risk of bias and promote study rigour, the following steps were followed: (i) we documented an a priori methodology; (ii) two or more members of the research team conducted data analysis; (iii) accurate recording and transcription of audio data; (iv) only health professionals with relevant experiences of hospital falls prevention were recruited; and (v) emerging themes were linked back to relevant participant quotes [37, 38].

The study was approved by the La Trobe University Human Research Ethics Committee (HEC19373) and was registered in the Australian New Zealand Clinical Trials Registry (ACTRN12620000033943).

## Results

### Participant characteristics

Four focus groups were conducted, with a total of 23 participants. Two focus groups comprised rehabilitation nurses (n = 7) and the other two groups included allied health professionals (n = 16) such as physiotherapists (n = 9), occupational therapists (n = 4) and allied health assistants (n = 3). The average years of clinical experience for the health professionals was 9.6 (SD 7.56, range 1–30). The average duration of the focus groups was 50.6 minutes (SD 8.4). Table 2 summarises participant characteristics.

**Table 2:** Health professional participant demographics.

Participant characteristics	Participants (n = 23)
Mean age, y (SD)	34.1 (9.99)†
Gender	
Female	17
Male	6
Post-graduate qualification*	3
Undergraduate qualification (Bachelor degree)	15
Other qualification**	5
Type of clinical experience†	
Acute	15
Sub-acute	20
Other‡	6

* Includes Masters of Rehabilitation Gerontology, Graduate Diploma in Rehabilitation, Postgraduate Applied Science;

** Includes Diploma of Nursing, Certificate IV;

† Data from one participant missing;

‡ Outpatient, community, clinics

Two overarching themes were identified from the qualitative analysis. The first pertained to perceived barriers to providing and implementing patient falls education that were: (i) sub-optimal systems for falls education for patients and health professionals; (ii) perceived patient-related barriers to evidence-based falls education; and (iii) limited interprofessional communication about patient falls. The second related to health professional views on how best to facilitate enablers to provide patient falls education. This incorporated sub-themes on: (i) facilitating interprofessional teamwork and collaboration; (ii) implementing strategies to increase patient empowerment; and (iii) selecting effective modes of education delivery.

### Theme 1: Barriers to the provision of patient falls education

#### 1.1 Sub-optimal systems for falls education for patients and health professionals

Many participants (n = 14) identified barriers at an organisational level, citing inconsistencies in clinical practice. These included variations in the content and design of falls education programs, differences in documenting falls and falls risk, and disparities in falls prevention education and communication between hospital sites, professions, and patients (Box 1). A third of participants viewed communication about falls prevention as being inconsistent between hospital sites. One example was differing organisational policies where patients were given non-slip socks to prevent falls in an acute setting yet encouraged to wear sturdy footwear to prevent falls in a sub-acute setting. One clinician highlighted that, due to hospital policy, falls prevention strategies were often broadly implemented using standard methods instead of being individualised to the needs of patients (Box 1).

**Box 1. Sub-optimal systems for falls education for patients and health professionals**

“Often there’s discrepancies between what we’ve documented, what’s documented on the progress notes, and then what’s documented by non-therapy staff as to what the patient’s mobility is” *Focus group 1*, *participant 1 (physiotherapist)*

“Communication is definitely something that fails because of time. We’re all under a lot of pressure and the same for nursing, and at certain times of the day they’re under more pressure.” *Focus group 4*, *participant 23 (allied health assistant)*

“We assess a patient for admission…there is a lot for us to document in an admission … along with doing observations…There’s a lot to it and…they’ll have another five patients. So, you need to look after the other five patients, but then also sit down and spend time educating the patient on falls or discussing falls or–when would you have the time?” *Focus group 3*, *participant 16 (nurse)*

“Patients who clearly aren’t mobilising, like one of the patients on my ward at the moment has just had a fall but has never been referred to physio [for education]. But in all the notes there’s been supervision even though she was at home alone. Or inconsistent documentation about what the mobility status is.” *Focus group 1*, *participant 9 (physiotherapist)*

“If someone’s admitted through ED at a weekend, for example, if they’ve come from a nursing home sometimes it’s not very accurate what their baseline is. And it’s just a case of phoning up the nursing home and asking the staff what exactly their status is before you go and assess them. But they may not have been done and then staff might have tried to get them up in the meantime” *Focus group 1*, *participant 10 (physiotherapist)*

“We’re supposed to do the FRAT but I’m not following that FRAT score myself anymore. I’m just using my clinical judgement whether this patient’s low, moderate, or high falls risk” *Focus group 4*, *participant 20 (occupational therapist)*

“A universal policy that people, every staff member adheres to patients walking with covered shoes with a good grip. I think that’s probably what we should be doing but I’m not sure if that’s universally adopted or implemented in a really clear way across nursing, OT, physio” *Focus group 4*, *participant 18 (physiotherapist)*

“We no longer give out grip socks here, but they’ll come from the acute hospitals with grip socks.” *Focus group 2*, *participant 11 (nurse)*

“You had patients who had shoes, who could clearly put those shoes on but because of the policy they were almost not forced, but they were pressured into wearing grip socks and these patients are about 30 years of age, 45 years of age. And it was just to tick a box in the campaign.” *Focus group 1*, *participant 1 (physiotherapist)*

“they [physiotherapists] don’t run any education. They assess a patient with how safe they are with mobility… But the occupational therapist is looking at the bigger picture for education and discharge planning” *Focus group 3*, *participant 16 (nurse)*

Another barrier was a perceived lack of capacity, time, or staff resources to deliver evidence-based falls prevention education. A third of the participants reported that discrepancies existed in communication between professions and between patients. This variability was sometimes associated with time constraints or staffing ratios. Another organisational barrier related to documentation, which was considered to be time consuming. It was also reported that falls prevention strategies were inconsistent across settings. Examples of quotes on these themes are presented in Box 1.

#### 1.2 Perceived patient-related barriers to evidence-based falls education

Almost half of all participants identified patient-related factors as a barrier to providing falls education (Box 2). Some health professionals reported a lack of insight by patients about their falls risk which limited their receptiveness to falls prevention education. Health professionals noted that, due to the desire to maintain dignity or independence, or to not “cause trouble” for staff, some patients did not follow recommended falls education advice. This could result in patients avoiding using the call bell, not waiting for assistance, or avoiding using recommended mobility aids. Systemic issues and organisational barriers could also contribute to slow call bell responses. Five of the health professionals reported their view that some patients choose to disregard the falls education due to their beliefs, situation or personality (Box 2).

**Box 2. Perceived patient-related barriers to evidence-based falls education**

“…That perception that they don’t want to disturb the nurse. They don’t want to press the buzzer” *Focus group 2*, *participant 13 (nurse)*

“They’re not wanting to give up their independence. So, their reluctance to pull the bell because they’ve been independent. They feel like a burden. They’re not wanting to pull the bell so they might attempt to–even though they might not be safe” *Focus group 1*, *participant 2 (physiotherapist)*

“Sometimes it’s hard to tell if language barrier … is an issue … in the moment or if it’s a personality thing, or a cultural thing. Again, sometimes men just don’t seem to like being told by women what to do. … Sometimes women don’t like to be told.” *Focus group 4*, *participant 22 (allied health assistant)*

“They might not realize that they are at risk of having a fall, so they might be just really confident. Their mobility has been fine at home before hospital. They don’t realize that things have changed…I think just lack of insight probably” *Focus group 3*, *participant 17 (nurse)*

“Some of the younger ones that can have really poor mobility think they’re a lot better than what they are. And they don’t want to use those because they think they’re not old enough for aids and those types of things” *Focus group 1*, *participant 8 (occupational therapist)*

“They’ve [patients] definitely been given that information time and time again, and have chosen not to take it on-board…then we’re left at that kind of path of where do we go from here. We’ve given them as much information as we can, we’ve reiterated, and they definitely have the same outcome time and time. So, it’s one of things that how much can you really get a patient to do if they’re just not willing to participate with your instructions.” *Focus group 1*, *participant 5 (physiotherapist)*

“There’s been a few that have fallen like that…they want to go to the toilet and…they’ll get embarrassed if they do it in their bed or their seat” *Focus group 1*, *participant 8 (occupational therapist)*

#### 1.3 Limited interprofessional communication about patient falls

A recurring theme was a perceived shortfall in interprofessional communication and health professional collaboration (Box 3). Over half of the participants reported that breakdowns in communication between professions was a major barrier to providing patient falls education. These breakdowns appeared to occur in both verbal and written communications. Two thirds of participants reported discrepancies in documentation about falls in medical records and other hospital documents. Of these participants, 6 allied health professionals spoke about the inconsistencies between health professional groups in documentation related to the functional and mobility status of patients. Two other allied health professionals reported differences in documentation between nursing shifts. The participants also noted discrepancies in written documentation between professions or reported a lack of collaborative discussion about falls risk and mitigation at handover. Three allied health professionals felt that some staff overlooked or disregarded their written recommendations about falls prevention. Two nurses held the view that formal falls education was the role of allied health professionals. One physiotherapist advised that all staff should be responsible for responding to patient needs to reduce their falls risk. This shortfall in teamwork and role clarification was viewed as a barrier to providing falls education (Box 3). Several clinical-related attitudinal factors were also highlighted by health professional participants. There was a perception of an over-reliance on processes that could lead to complacency. Two participants felt that completing forms could become routine and limit clinical judgements about the needs of individual patients (Box 3).

**Box 3. Limited interprofessional communication about patient falls**

“I don’t think the progress notes are necessarily followed. That’s the legal communication document, but people often refer to their own handovers and that interpretation of what’s written in the progress notes is not always [correct]… they’ve got their own interpretation of it.” *Focus group 1*, *participant 1 (physiotherapist)*

“We tell them [patient] that they should be encouraged to be independent as part of their physical conditioning being in a hospital. But then you read the nursing notes that say … ‘maximum assistance provided, or full assistance provided’” *Focus group 4*, *participant 21 (occupational therapist)*

“The patient is made aware of what they can and what they should ideally be doing. But it’s not necessarily always followed. And sometimes that information’s not handed over to the staff over the course of the day” *Focus group 1*, *participant 1 (physiotherapist)*

“Another element of why falls happen is the communication between the documentation and the staff. Because there can be just verbal communication from one staff member–I’m referring to nursing staff–at the end of a shift and then there can just be a little bit of miscommunication” *Focus group 1*, *participant 5 (physiotherapist)*

“I think we all know it [falls education], and we just leave it to the physios to do” *Focus group 2*, *participant 11 (nurse)*

“I was highlighting to everybody in the office that…everyone is capable of responding to that falls alarm. If that person had a fall and the research says that they may die within the next two years, that’s all our responsibility.” *Focus group 1*, *participant 9 (physiotherapist)*

“They have to go through it [falls risk FRAT form] and they have to tick all the things like age and medications…But I don’t know if it just becomes: “we need to do this form for admission, tick all the boxes” … whether it’s actually triggering a thought about it.” *Focus group 1*, *participant 9 (physiotherapist)*

“Some clinicians are very black and white, just based on the numbers, hence, you’re a high falls risk when they’re actually moderate. … I think it’s better to utilise your own clinical judgement and make that call” *Focus group 4*, *participant 20 (occupational therapist)*

### Theme 2: Enablers to the provision of patient falls education

#### 2.1 Facilitating interprofessional teamwork and collaboration

More than a third of participants identified the value of an interprofessional approach to facilitating falls prevention education. Nurses and allied health professionals advised that interprofessional teamwork facilitated consistent and frequent patient education. They expressed a view that, with effective communication, health professionals could address falls risk factors in a more united and holistic manner and have a collective approach to consistent falls education. Three allied health professionals advised regularly consulting nurses and other health professionals about the patient’s functional status to form a holistic view of their needs. Two nurses reported that they worked with the multidisciplinary team to identify falls prevention strategies for complex patients. This collaborative practice approach promoted collective responsibility and patient-centred care to ensure that individual patient needs were addressed by the team (Box 4).

**Box 4. Facilitating interprofessional teamwork and collaboration**

“Falls needs to be taken as a holistic view where the team gets involved around the patient’s fall. This is not targeted at any one type of clinician but as the team looking after the patient. If they’ve come with a fall that it should trigger a pathway of thinking or risk assessment about this patient’s holistic presentation” *Focus group 1*, *participant 9 (physiotherapist)*

“To me it’s the communication. I speak to the nurses, what do they [patients] do…what are they doing and that’s where falls education happens as well.” *Focus group 4*, *participant 20 (occupational therapist)*

“Here we’re lucky with all our allied health staff, because…they will reiterate what we say.” *Focus group 2*, *participant 11 (nurse)*

“It’s about us nursing staff communicating with the team and the doctors and the allied health to see if there is something that we’re not using at this point and see what other options we have for that patient.” *Focus group 3*, *participant 16 (nurse)*

“Sometimes during handover there would be a highlight of, “Look, you know, we’re not happy with the mobility of this patient.” Physio will be asked to assess, and then we’ll wait for their assessment, and then it has to be handed over. And everybody that is on that shift has to know, just in case.” *Focus group 2*, *participant 15 (nurse)*

#### 2.2 Implementing strategies to increase patient empowerment

The health professionals identified patient empowerment as a strong enabler to education. More than two thirds recommended a focus on empowering patients to improve the uptake of falls education. They suggested this could be achieved by individualising the education, encouraging patients to reflect on their falls risk and ensuring the education was relevant to the patient’s situation. Health professionals felt that this allowed for deeper learning and an increase in an adoption of falls prevention strategies. In addition, there was agreement that education should be continuous throughout a patient’s stay. The majority felt that empowering patients across the continuum of care could lead to improved outcomes (Box 5).

**Box 5. Implementing strategies to increase patient empowerment**

“Empowering them with the knowledge of why, not just this is what’s happening.” *Focus group 4*, *participant 23 (allied health assistant)*

“I think they need to know why they’re a falls risk because then they can–if they understand it, they’re more likely to comply because they will know why rather than just, ‘I’m a falls risk. I don’t agree with that. I’m just going to walk.’” *Focus group 3*, *participant 17 (nurse)*

“The campaign’s very much like a very broad brush that’s used…the next campaign it shouldn’t take that approach, it should be based on what’s needed for that particular patient.” *Focus group 1*, *participant 1 (physiotherapist)*

“Sometimes following up and just asking them how they felt about it [education] afterwards and things like that…just asking questions after it and seeing how they respond, and judging that way.” *Focus group 1*, *participant 7 (allied health assistant)*

“We get them to identify the falls risks and then we have another discussion about what can we do to fix that risk… So they’ll come up with ideas, so they’re thinking about it more than just us telling them.” *Focus group 1*, *participant 8 (occupational therapist)*

“It depends on therapists’ clinical judgements whether these patients will benefit from falls prevention groups or whether just one on one… Behaviour, personality, whether they actually interact in discussions. I think it’s really quite personal” *Focus group 4*, *participant 20 (occupational therapist)*

“I think that the reiterating of knowledge throughout their entire stay through rehab…constant falls prevention education is really beneficial for patients if we’re incorporating it into everything we’re doing” *Focus group 1*, *participant 4 (occupational therapist)*

“I think the content of the education’s one thing, I think you need to observe how they’ve taken the education on… You’ve got to actually observe and then document implemented safe technique, so that you’ve documented that you’ve not only imparted the education but that they’ve changed what they were doing in order to change” *Focus group 4*, *participant 18 (physiotherapist)*

#### 2.3 Selecting effective modes of educational delivery

The way in which education was delivered was perceived to be an important facilitator for effective falls mitigation. This included the nature of the messaging about falls prevention and the methods that health professionals used for falls education delivery. Over two thirds of participants reinforced the importance of consistent and frequent provision of education across and within professions, and throughout the continuum of care. Ten supported verbal reinforcement of falls education, believing that reiterating the education emphasised its importance as well as encouraging knowledge retention.

There were different suggestions for methods of delivery. One third of participants recommended making the education interactive to increase engagement with patients. Some suggested asking questions to encourage self-reflection and others recommended group education to share experiences and learn from each other. Another mode of education was the use of falls risk reminders. A third of participants felt that these reminders could assist in reinforcing the education. Six participants reported that involving families to reiterate falls education had beneficial effects. The families were then able to reinforce the education in hospital and at home, encourage adherence to falls prevention strategies and monitor patients for risk-taking behaviours (Box 6).

**Box 6. Selecting effective modes of educational delivery**

“Every time I see a patient, I mention wearing their shoes, so always ask them to wear proper shoes.” *Focus group 4*, *participant 19 (physiotherapist)*

“Some of the patients have seen the falls DVD two or three times and they go, “Not this again.” But then when I talk to them later, they pick up something else that they haven’t picked up on the other ones…Familiarity breeds more knowledge when they see it a few times and they pick up different things, and it sinks in. They see it once and it’s gone.” *Focus group 1*, *participant 8 (occupational therapist)*

“More of an interactive coaching interview, you know how you were saying that people come up with the ideas themselves they’re more likely to implement them or be responsive to them. So rather than just giving an interview and telling the person maybe making them reflect on their own current practices or talk about previous falls, what maybe could have led to that not happening. More coaching” *Focus group 1*, *participant 9 (physiotherapist)*

“We run a patient falls group and I’ve found…smaller patient groups it’s much more interactive. They actually share their experience about falls.” *Focus group 4*, *participant 20 (occupational therapist)*

“Let them see the falls prevention DVD and then we’ll talk about what they saw, what do they recall, and talk about the broader aspects of it as well” *Focus group 1*, *participant 8 (occupational therapist)*

“if someone’s got a supervision tag then every person who sees that patient can reinforce the same message around the guidelines that they’re supposed to be adhering to…maybe in the middle of the night they will call the nurse before they get up and go to the loo. … So maybe it enables more consistency across the staff." *Focus group 4*, *participant 18 (physiotherapist)*

“some, or most of our patients have either short-term memory loss, so if the family is there, we educate the family…we have an hourly round, so we find later on we iterate again, because they already forgotten.” *Focus group 2*, *participant 13 (nurse)*

“I think talking to family members too. And they’ve probably got a better idea of how they’ve been going at home, whether they’ve been falling at home. Talking about history. If your family member’s there, it’s usually a lot better to get an idea of what they’ve been like mobility-wise at home… and then including the family member when we’re talking about falls prevention, things to do at home.” *Focus group 1*, *participant 8 (occupational therapist)*

## Discussion

Health professionals have a key role in mitigating hospital falls and delivering evidence-based patient education [39, 40]. Our focus groups showed that nurses and allied health professionals are usually aware of the enablers and barriers to providing falls education. They can also generate different ideas on how best to facilitate patient falls education. Nevertheless, there were systemic issues and variability in practice that compromised their ability to consistently implement evidence based, patient-focussed, inter-professional falls prevention education. In addition, health professionals sometimes consider themselves to be the experts in falls prevention and there remains scope for further consumer engagement and co-design of education programs [1, 9].

Inconsistencies in clinical practice was a major barrier, as with previous reports [41–43]. Heng et al. [44] reported inconsistencies in falls education, content and delivery which limited the ability of patients to understand their risk of falling during an admission. Time constraints and lack of staffing capacity and resources were other challenges, in agreement with Ackerman et al. [45], Keyworth et al. [43] and Svavarsdóttir et al. [25]. There was often over-reliance on processes and forms which can take the focus away from clinical judgement [46].

Another area that health professionals identified for improvement was the follow up of patients after the initial provision of falls education. Health professionals recognised that repeated messaging was needed, although they cited several barriers to doing so and highlighted the complex nature of translating evidence-based knowledge into practice [47, 48]. Clinician follow-up has been shown by Berger et al. [49] and Beagley [50] as one way of reinforcing patient education, together with visual reminders and family involvement in reinforcing falls education messages.

Another barrier to providing falls prevention education was lack of communication between different health professionals. Lee et al. [41] reported that inconsistencies in education and communication can undermine the message of falls prevention and increased falls risk. Health professionals who provided hospital falls prevention education in the current study also identified inconsistencies between health professional assessments of mobility that increased falls risk. Inconsistent instructions to patients have likewise been shown by Hill et al. [51] to increase falls risk. Discrepancies in documentation and poor clinical handover practices can also be associated with adverse events [52, 53], including an increase in falls [54, 55]. In agreement with our focus groups, Foronda et al. [56] advised that interprofessional communication is paramount in ensuring safe patient outcomes.

Limited collaboration and teamwork between professions were further barriers to patient falls education. Interprofessional collaborative practice can improve patient outcomes and elevates health organisations and systems [57–59]. Interprofessional collaborative practice fosters a climate of safety and improved clinical practice [60–62]. Interprofessional education, where two or more professions learn with, from and about each other [63], can be a way to foster teamwork, collaboration, communication and patient-centred care [57, 64, 65]. McKenzie et al. [66] reported that interprofessional education resulted in improved falls screening and prevention. Wheeler et al. [61] demonstrated that evidence-based interprofessional education for falls prevention had a positive impact on the clinical practice of physiotherapists and occupational therapists. Health professionals often face challenges when implementing interprofessional education, such as having separate professional cultures [67], logistical barriers [68], limited shared resources in training [69], and varying clinical practice demands [70]. By ensuring consistency of patient education content across professions through interprofessional collaborative practice and interprofessional education, the message of falls prevention can be reinforced [71, 72].

Patient-centred factors also need to be taken into consideration. Patients may not always be aware of their falls risk or changes in mobility despite receiving falls education [73, 74]. They can also have a fear of embarrassment or a reluctance to burden staff, or have a false sense of security that adversely affects adherence to recommended strategies [22, 75]. According to Haines et al. [76], this can result in patients taking unnecessary risks which increase falls. Empowering patients to prevent their own falls across the continuum of care and within routine activities of daily living was a key recommendation. Involving patient representatives in the co-design and implementation of interventions and making education relevant, interactive and individualised allows for patients to better engage with the health professional and the education [77, 78]. Similar health behaviour changes through patient empowerment have also been identified by Joseph-Williams et al. [79] and Michie et al. [80]. As with Heng’s trial on patient perspectives [74], the health professionals in the current study identified frequent and consistent provision of patient education to be a key determinant of hospital falls. Likewise, in the design of future interventions, there is a need to consider initiatives that focus on improving collaboration and teamwork, along with being more patient-centred and tailoring educational materials to individual needs.

The modes of educational delivery were perceived to be a key enabler for effective falls education. In line with participant views, group education [81, 82], interactive education [83], and encouraging self-reflection [84] have been found to improve engagement and reinforce learning. Involving families when delivering education can also lead to better patient outcomes [85]. Findings about the delivery of education are a key determinant of the success of falls prevention programs in hospital [4]. However, in this study there was a lack of focus on the quality of educational design. According to Heng et al. [9], the quality of educational design can be linked with improved hospital falls outcomes. As shown by Shaw et al. [1], educational design is a powerful tool to influence the success of falls prevention training for health professionals and patients alike.

There were several limitations of this study. Primarily, the focus groups were limited to English speaking participants in hospitals in one state of Australia. Most participants were based in sub-acute hospital wards and the findings might not be generalisable to other populations or other countries. Also, this study focused on hospital education programs and might not be generalisable to outpatients or community populations. There are inherent limitations of using focus groups as a means for collecting data. For example, health professionals might have varied levels of employment in their institution and power imbalances can occur within groups [86]. These factors need to be considered in the design of future studies. There is also a need to further explore the growing body of work about dignity of risk, particularly in respect to falls management in hospitals. The strengths of this study included representation from different health professions and the high level of expert falls contextual knowledge of participants. Trustworthiness was strengthened by experienced moderators who were also experts in the field.

## Conclusion

Health professionals working in hospital settings recognised enablers and barriers to providing patient falls prevention education. Managing inconsistencies in clinical practice, communication barriers and professional silos were hurdles. Enablers included carefully designed interactive education resources tailored to individual needs of patients, as well as implementation of patient-centred hospital processes to empower the patients to gain knowledge. Systems to foster collective responsibility amongst health professionals to create a culture of vigilance regarding preventing hospital falls were also key.

## Supporting information

S1 Checklist Consolidated criteria for reporting qualitative studies (COREQ): 32-item checklist. (DOCX)
